# Advantages and Limitations of Direct PCR Amplification of Bacterial 16S-rDNA from Resected Heart Tissue or Swabs Followed by Direct Sequencing for Diagnosing Infective Endocarditis: A Retrospective Analysis in the Routine Clinical Setting

**DOI:** 10.1155/2016/7923874

**Published:** 2016-03-24

**Authors:** Daniela Maneg, Janina Sponsel, Iris Müller, Benedikt Lohr, John Penders, Katharina Madlener, Klaus-Peter Hunfeld

**Affiliations:** ^1^Institute of Laboratory Medicine, Microbiology and Infection Control, The Northwest Medical Centre, 60488 Frankfurt am Main, Germany; ^2^CAPHRI School for Public Health and Primary Care, Department of Medical Microbiology, Maastricht University Medical Center, 6200 MD Maastricht, Netherlands; ^3^Kerckhoff Klinik, 61231 Bad Nauheim, Germany

## Abstract

Infective endocarditis (IE) is a life-threatening disease that is associated with high morbidity and mortality. Its long-term prognosis strongly depends on a timely and optimized antibiotic treatment. Therefore, identification of the causative pathogen is crucial and currently based on blood cultures followed by characterization and susceptibility testing of the isolate. However, antibiotic treatment starting prior to blood sampling or IE caused by fastidious or intracellular microorganisms may cause negative culture results. Here we investigate the additional diagnostic value of broad-range PCR in combination with direct sequencing on resected heart tissue or swabs in patients with tissue or swab culture-negative IE in a routine clinical setting. Sensitivity, specificity, and positive and negative predictive values of broad-range PCR from diagnostic material in our patients were 33.3%, 76.9%, 90.9%, and 14.3%, respectively. We identified a total of 20 patients (21.5%) with tissue or culture-negative IE who profited by the additional application of broad-range PCR. We conclude that broad-range PCR on resected heart tissue or swabs is an important complementary diagnostic approach. It should be seen as an indispensable new tool for both the therapeutic and diagnostic management of culture-negative IE and we thus propose its possible inclusion in Duke's diagnostic classification scheme.

## 1. Introduction

In spite of improvements in the diagnostic and treatment, infective endocarditis (IE) remains a serious, life-threatening disease [1–4] with an unchanged mortality rate up to 30% and an incidence of 2 to 6 per 100,000 individuals per year over the last 30 years [5]. The stagnation in mortality and incidence can be ascribed to the progressively evolving predisposing factors [6] leading to a change in the epidemiology and etiology of IE, thereby resulting in worsening of the individual course of IE [1, 5, 6]. Particularly noteworthy is the general increase of reported health care associated IE [7] with a potentially higher proportion of microorganisms with a potentially complex resistance pattern. Especially for* S. aureus* bloodstream infections, IE is referred to as most frequent complication and occurs in 40% of patients without heart disease [8]. Still, the primary sites of infection are native heart valves, but individuals with implanted mechanical devices such as valvular prostheses, pacemakers, or implantable defibrillators are increasingly involved [9–11].

Early diagnosis of IE and rapid identification of the causative pathogen are crucial for providing a specific and efficient antibiotic therapy and improvement of the patient [5, 12–15]. Generally, in IE patients, blood cultures are frequently positive [16]. Nevertheless, such cultures can remain negative in 2.5% to 31% of all cases of IE, depending on causative agent and the clinical circumstances [2, 17, 18]. Blood cultures typically remain negative due to antibiotic therapy prior or concurrent to blood sampling (~50% of cases), due to infection by fastidious organisms such as nutritionally variant streptococci, fastidious Gram-negative bacilli of the HACEK group (*Aggregatibacter aphrophilus*, former* Haemophilus aphrophilus* and* H. paraphrophilus*;* Aggregatibacter actinomycetemcomitans*, former* Actinobacillus actinomycetemcomitans*;* Cardiobacterium hominis*;* Eikenella corrodens*;* Kingella kingae*),* Brucella* spp. (in endemic areas), and fungi or due to infections by intracellular bacteria such as the zoonotic agents* Coxiella burnetii* and* Bartonella* and* Tropheryma whipplei* [5]. In such cases, negative cultures may significantly delay diagnosis and effective treatment which possibly negatively impacts on the outcome of the patient [17].

These obvious drawbacks of conventional techniques clearly impair the current criteria used for clinical diagnosis of IE and call for adjunctive diagnostic methods increasing the sensitivity for more rapid pathogen recovery and identification. This is why in such cases of blood culture-negative or clinically questionable IE, culture-independent, new molecular diagnostic techniques such as broad-range polymerase chain reaction (PCR) with subsequent DNA-sequencing from heart valve specimen have gained increasing attention and were shown to be useful in the detection and classification of bacterial DNA in several recent diagnostic studies [19–28]. Besides detecting organisms that are missed by culture-dependent methods, PCR-based methods also allow exact identification of the causative organism and the identification of new hitherto unknown pathogens causing IE even if the patient is already under antibiotic treatment [29]. These possibly beneficial features of broad-range PCR for the diagnosis in such cases have even led to the proposition of PCR positivity as a major diagnostic criterion for IE [14, 19, 30]. However, further research is clearly required until PCR-based methods become accepted as routine diagnostic tool [2, 9].

The aim of the present study was to investigate the additional diagnostic value of broad-range PCR targeting the 16S ribosomal DNA in heart tissue or swabs in patients with confirmed, suspected, or possible IE in a routine clinical diagnostic setting. We hypothesized that the use of broad-range PCR amplification followed by direct sequencing might increase the number of identified causative pathogens in cases with culture-negative IE and in antibiotically pretreated individuals thereby possibly improving the rapidity of diagnosis in such patients also in the routine diagnostic setting.

## 2. Material and Methods

### 2.1. Study Design and Patients

**Figure 1:**
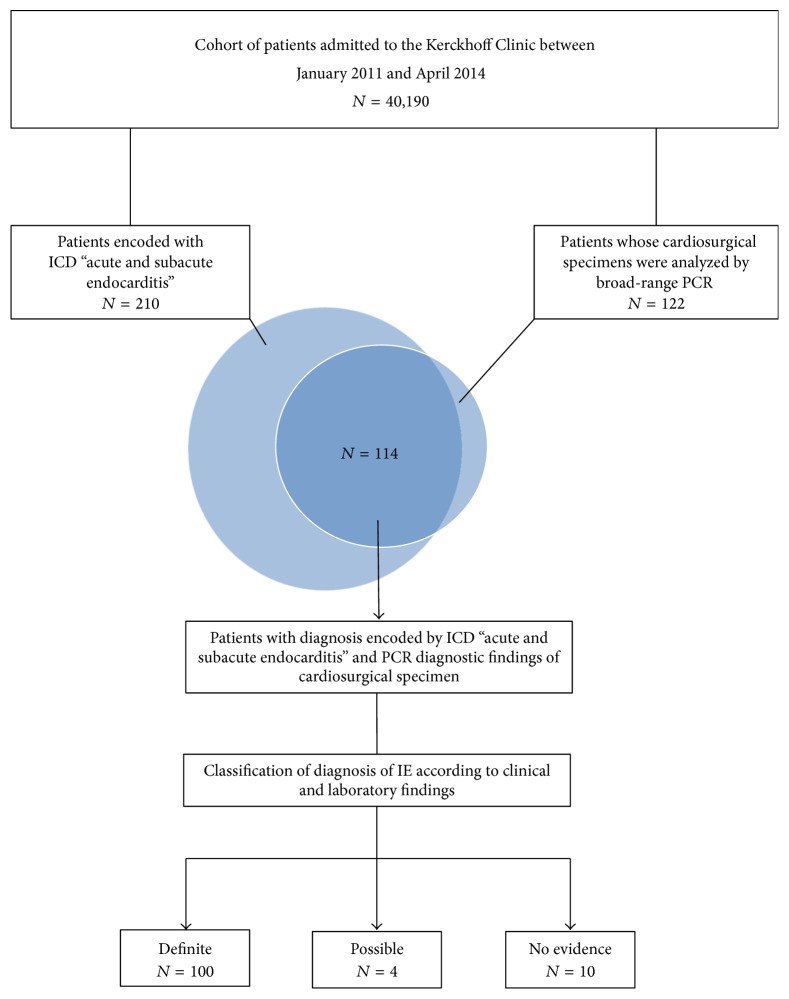
Overview of the assortment of the study group. Workflow scheme for the assortment of patients according to the criteria described in detail in Section 2.

For our retrospective observational study, we identified all patients admitted to Kerckhoff Clinic in Bad Nauheim (Germany), between 2011 and 2014 (Figure 1). This cohort was then subjected to further in-depth computer-assisted analysis identifying all patients admitted with a diagnosis of suspected or definite infective endocarditis encoded by the ICD-10-GM (International Classification of Diseases, 10th Revision, German Modification) for “acute and subacute endocarditis” (I.33.-) and undergoing cardiosurgical treatment. We also looked for all patients subjected to diagnostic testing after admission to Kerckhoff Clinic by broad-range PCR amplification and direct sequencing from cardiosurgical specimens as performed at the Institute of Laboratory Medicine, Microbiology and Infection Control at the Northwest Medical Centre in Frankfurt/Main (Germany) during the same time period. In combining both selectors, all patients were identified with both an encoded diagnosis of “acute and subacute endocarditis” and a diagnostic test result of broad-range PCR and direct sequencing from cardiosurgical specimens for further investigation (Figure 1). Medical charts of all patients of this study group were then retrospectively reviewed and patients were included in the study if both the microbiological findings or broad-range PCR and the corresponding tissue or swab culture of cardiosurgical specimens were available.

### 2.2. Definition of Diagnosed IE

For the purpose of this study, patients of the study group were divided into three different categories (“definite,” “possible,” and “no evidence for” IE; Figure 1) according to some modification of Duke's criteria for the diagnosis of IE [31] and including all available data from the medical charts at Kerckhoff Clinic as follows: definite IE: a typical valve histopathology (considered as gold standard) in addition to 2 or more findings indicating IE in any of the following diagnostic procedures is present: echocardiography, preoperative BC (preBC), and typical intraoperative macroscopic aspect; possible IE: there is negative valve histopathology or evidence for IE resulting from one of the above-mentioned diagnostic procedures only, but there is a positive result upon PCR and/or tissue or swab culture from valve tissue after surgery; no evidence for IE: only one of the above described procedures provided evidence for IE but PCR, conventional culture from valve tissue after surgery, and histopathology proved negative (Figure 1).

### 2.3. Surgical Material

Tissue specimens or swabs from intraoperatively excised cardiac tissue were taken during cardiosurgical treatment at Kerckhoff Clinic in Bad Nauheim according to local standard surgical and diagnostic procedures, and specimens for microbiological analysis were then delivered to the Institute of Laboratory Medicine, Microbiology and Infection Control at the Northwest Medical Centre in Frankfurt/Main (Germany) for microbiological analysis performed under standard conditions.

### 2.4. Molecular Methods

Bacterial DNA was isolated from the intraoperative excised specimens by means of the MagNA Pure Compact System (Roche Diagnostics, Mannheim, Germany). For the amplification of the 16S-rDNA with a GeneAmp PCR System 2700 or a Thermal Cycler 2720 (Applied Biosystems, Darmstadt, Germany), the universal 5′ primer 285 (gag agt ttg atc ctg gct cag, position 9–29 within gene according to 16S-rDNA of* Escherichia coli*) and the universal 3′ primer 243r (acg agc tga cga cag cca tg,* E. coli* position 1073–1054) (both primers synthesised by TiB Molbiol, Berlin, Germany) were used [32]. From visible UV-bands PCR products were purified with the QIAquick PCR Purification Kit (50/250) (QIAGEN, Hilden, Germany). Sequencing was carried out using a BigDye Terminator v.1 or alternatively using a BigDye Terminator v3.1 (Applied Biosystems, Darmstadt, Germany) according to the manufacturer's instructions. Analysis of the sequencing products was carried out using ABI 3500 Dx Sequencer (Applied Biosystems, Darmstadt, Germany). For the identification of the pathogens, the resulting sequences were then compared with reference sequences obtained from the NCBI database using BLAST (Basic Local Alignment Search Tool, http://blast.ncbi.nlm.nih.gov/).

## 3. Results

### 3.1. Assortment of Patients

In our observational retrospective study, we identified a total of 40,190 patients admitted to Kerckhoff Clinic in Bad Nauheim between January 2011 and April 2014. A total of 114 patients with suspected or diagnosed IE who underwent cardiosurgical treatment during this time period were included in our study. One hundred patients had definite IE and 4 patients had possible IE according to our above defined criteria. For 10 patients there was no evidence for the clinically suspected diagnosis of IE. The patients with definite or possible IE (*n* = 104) were included in our further analysis whereas the 10 patients with no definite evidence for IE were excluded and used for the computation of sensitivity, specificity, and positive and negative predictive values only. The incidence of IE in our cohort of admitted hospital patients was found to be 259 per 100,000 patients/year.

### 3.2. Description of Patients and Surgical Material

Out of 104 selected patients with possible or definite IE, 75 were men and 29 were women (F/M ratio: ~1 : 3) with a mean age of 66.0 ± 12.6 years. The primary sites of infection were native heart valves (64.6%) with 37 aortic (50.7%), 34 mitral (46.6%), and 2 tricuspid (2.7%) valves. 30.1% were prosthetic valves (aortic valve, *n* = 28, 82.4%; mitral valve, *n* = 5, 14.7%; and tricuspid valve, *n* = 1, 2.9%) whereas 5.3% showed infected cardiac devices (*n* = 6) (Table 1). Nine of the 104 patients suffered from two infected heart valves or an infected valve together with an infected cardiac device (aortic/mitral valve, *n* = 7; prosthetic aortic/mitral valve, *n* = 1; mitral valve/cardiac device, *n* = 1) according to the diagnosis documented in the medical charts (Table 1).

### 3.3. Comparison of PCR Results with Clinical Findings

**Figure 2:**
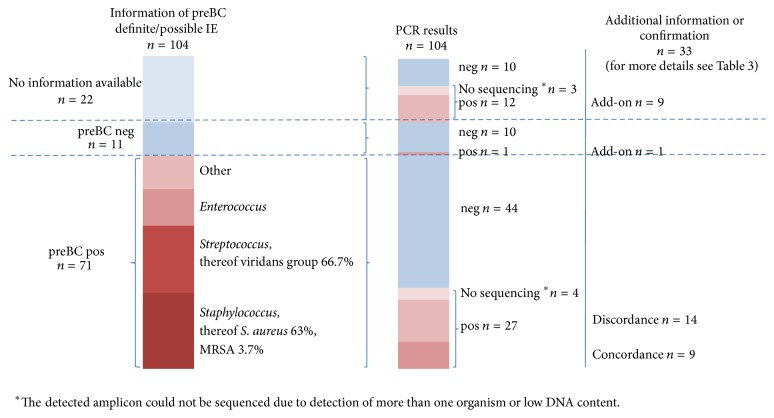
Comparative presentation of PCR results with preoperative blood culture. Bars represent on a percentage basis the distribution of preoperative blood culture results (*preBC*), correlated with the results of PCR followed by direct sequencing. Blue shaded bars represent negative results or no available information. Red shaded bars represent a positive result in blood culture or PCR amplification. In the column on the right hand side further information is provided. The results of direct sequencing are classified according to either a concordance or discordance in comparison to the results of blood culture or as add-on information in case of a negative or not available result. An in-depth detailed comparison is additionally shown in Table 3.

Bacterial broad-range PCR amplification was positive in 40 samples (38.5%) obtained from 104 patients with possible or definite diagnosis of IE. In 36 of 100 patients (36.0%) PCR results showed analytical agreement with the clinically defined diagnosis of definite IE. In 4 patients with possible IE that lacked clinical diagnostic evidence, broad-range PCR from valve tissue yielded a positive result supporting the clinical suspicion (Table 2, Figure 2).

In 33 of the 40 patients with positive PCR amplification (82.5%), sequencing of the amplicons was possible up to the species level and identified mainly streptococci and enterococci (both *n* = 9, 27.2%), followed by staphylococci (*n* = 8, 24.2%) and sporadically occurring pathogens (*n* = 7, 21.2%). The majority of the streptococcal sequences were identified as streptococci of the viridans group (*n* = 7, 77.8%), for example,* S. mitis* and* S. gordonii*. Among the staphylococci,* S. aureus* was identified most frequently (*n* = 4, 50%). Among the sporadically occurring bacteria,* Aerococcus urinae* (*n* = 1),* Anoxybacillus* spp.,* Bartonella quintana* (*n* = 1),* Gemella haemolysans* (*n* = 1),* Granulicatella adiacens* (*n* = 1), and* Propionibacterium acnes* (*n* = 2) were found (Table 3).

In the remaining 7 (17.5%) of the 40 patients with positive broad-range PCR, sequencing was not possible due to a mixture of DNA belonging to different organisms (*n* = 4, 57.1%) or due to low DNA concentrations (*n* = 3, 42.9%) in the sample. At least 33 of the 40 PCR-positive patients were treated with antibiotics preoperatively; for the remaining 7 patients these data were not available.

In 64 of 100 specimens, PCR amplification was negative despite the clinically confirmed diagnosis of IE in those patients. At least 60 of these 64 negative tested patients (93.8%) received antibiotics before surgery. For the 10 patients without clinical evidence for IE, PCR amplification was negative and further confirmed the absence of IE (Table 2).

### 3.4. Comparison of PCR Results with Preoperative Blood Cultures

Data of preBC were available for 82 of the 104 patients (78.8%) among which 71 were positive (86.6%) and 11 were negative (13.4%). Staphylococci were detected most frequently with 35.5% (*n* = 27), followed by streptococci (*n* = 24, 31.6%), enterococci (*n* = 13, 17.1%), and other (*n* = 12, 15.8%). On species level 63.0% were* Staphylococcus aureus* (*n* = 17) one of which was methicillin-resistant* S. aureus* (MRSA). 66.7% of the streptococci (*n* = 16) were represented by streptococci of the viridans group; for a detailed overview see Figure 2.

Of the 71 positive preBC, 27 were confirmed by positive broad-range PCR amplification results (38.0%). However, in 14 cases (52%) the pathogens identified differed between both techniques on genus and/or species level (see Table 3). In one patient where preBC identified two species of bacteria (*E. coli* and* Streptococcus mitis*), the presence of more than one species was confirmed by broad-range PCR which identified a DNA mixture in the sample. In the remaining 44 of the 71 blood culture-positive cases (62.0%), broad-range PCR was negative. 43 of these patients with blood culture-positive but PCR-negative results (97.7%) received preoperative antibiotics. Only 1 of the 11 blood culture-negative patients (9.1%) had a positive PCR result. In the remaining 10 patients with blood culture-negative results, PCR results were consistent. All of these patients were treated with antibiotics preoperatively (Figure 2).

### 3.5. Comparison of PCR Results with Results of Tissue or Swab Culture

Broad-range PCR was positive in 40 of 104 patients with definite or possible IE (38.5%) but at least 33 patients of those PCR-positive IE received antibiotics preoperatively. For the remaining 7 patients data about preoperative antibiotic treatment were not available (Table 2).

In 11 patients, broad-range PCR was performed exceptionally before having obtained a negative result of tissue or swab culture upon special clinical request. Seven of the culture-positive cases (63.6%) were concordant with a positive broad-range PCR, whereas 4 cases (36.4%) showed a negative PCR result. Among the cases with both a positive culture and PCR result, the identified pathogens differed among both techniques in 2 cases where broad-range PCR identified* Streptococcus gordonii* and* Streptococcus gallolyticus* whereas tissue or swab culture revealed* Staphylococcus epidermidis* and* Staphylococcus pasteuri* suggesting possible contamination of culture (Table 4). Nevertheless, these data were described only but not used for the computation of sensitivity, specificity, and positive and negative predictive values of broad-range PCR.

Of the 33 culture-negative cases (Table 5), the identification of the causative pathogens by broad-range PCR was possible in 26 specimens (78.7%) and revealed predominantly streptococci [*n* = 7, 21.2%], followed by enterococci and staphylococci [both *n* = 6, 18.2%]. For 7 specimens [21.2%], sequencing of the PCR products could not identify the etiology of the pathogens due to the presence of a DNA mixture or too low DNA concentrations in the sample.

### 3.6. Sensitivity, Specificity, and Positive and Negative Predictive Value of Broad-Range PCR

For the computation of the sensitivity, specificity, and positive and negative predictive values of broad-range PCR performed on tissue or swab specimens in tissue or swab culture-negative patients, all patients of the study (with definite, possible, and no evidence for IE) with a negative tissue or swab culture were included (*n* = 103).

Sensitivity and specificity of the broad-range PCR performed in cardiac tissue or swab specimens in tissue or swab culture-negative IE were 33.3% (23.8–44.1%; 95% interval) and 76.9% (46.2–94.7%), respectively, whereas the positive and negative predictive values of the broad-range PCR were 90.9% (75.6–98.0%) and 14.3% (7.1–24.7%), respectively (Table 6).

## 4. Discussion

Despite advances in diagnostic and therapeutic procedures, infective endocarditis remains a serious [1–3], life-threatening disease where long-term prognosis strongly depends on an immediate and optimized antibiotic treatment. Therefore besides an early establishment of the diagnosis, the rapid and correct identification of the causative microorganism is particularly important for the patient's prognosis [13, 33]. Currently, blood cultures remain the diagnostic mainstay for identification and species-specific characterization of IE-associated pathogens also because they offer the possibility of antimicrobial susceptibility testing for target directed antimicrobial treatment. If antibiotic treatment, however, is started prior to or concurrent to blood sampling or if IE is caused by slow-growing, fastidious or intracellular microorganisms, blood cultures may yield negative results [2, 18] which may lead to inadequate treatment and worsening of the patient. Here, molecular methods such as broad-range PCR amplification followed by direct sequencing of bacterial DNA performed in excised heart valve tissue hold promise in revealing the etiology of culture-negative IE. The broader diagnostic value of PCR for routine microbiological diagnostics, however, needs further evaluation until these techniques may become more generally accepted [13]. In this retrospective study, we, therefore, investigated the possible additional value of broad-range PCR amplification followed by direct sequencing performed in resected heart tissue or swabs for diagnosing IE in 104 patients with suspected or diagnosed IE in a routine clinical setting.

The incidence of IE in our cohort was found to be 2.6 per 1,000 patients per year which is much higher than reported in the general population (~2–6 per 100,000 individuals per year) [5] due to the fact that we focused on patients admitted to a specialized clinic for cardiac surgery. The patients included in our study still reflect the changing epidemiology of IE described in the literature [5] with a high mean age of 66.0 years. Also, men were affected more often than women (~3 : 1) and besides native heart valves (64.6%) we observed an increased number of infections of prosthetic valves (30.1%) and cardiac devices (5.3%) as the primary site of infection [11]. The number of infected prosthetic valves in our study is higher than described in recent investigations [5]. This may again be explained by the selection of our study population from individuals admitted for cardiac surgery which is indicated in case of progressive prosthetic valve endocarditis [34]. Relating to the microbial spectrum, all microorganisms identified corresponded to commonly known pathogens causing IE [16] except for one isolate of* Anoxybacillus* spp. detected by broad-range PCR and two identified by blood culture only (*S. alactolyticus* and* S. auricularis*).

Considering mainly clinical findings as the gold standard for diagnosing IE, the sensitivity and specificity of broad-range PCR performed in resected heart valve tissue or swabs to diagnose IE in heart valve tissue or swab culture-negative IE were 33.3% and 76.9%, respectively, whereas the positive and negative predictive values were 90.9% and 14.3%, respectively. In our study, broad-range PCR testing was only applied when tissue or swab culture from resected heart tissue remained sterile. Thus, for the evaluation of the accuracy of broad-range PCR in diagnosing IE, only patients with tissue or swab culture-negative IE were considered. This is probably why values of specificity, sensitivity, and positive and negative predictive values were somewhat lower than those reported in other studies (ranges: sensitivity: 41.2 to 96.0%, specificity: 95.3–100%, positive predictive value: 98.4–100%, and negative predictive value: 26.2–88.5%) [14, 19, 24, 25, 35]. Additionally, when comparing such values it should be kept in mind that not all of these studies were performed in a routine clinical setting. In contrast to classical prospective study conditions routine application may impair the elaborateness of preanalytical conditions and the management of practical procedures such as sampling and handling of the resected material which can negatively impact on the outcome. In this study, broad-range PCR was performed not only on resected heart tissue as the preferred analytical material but also on clinical swabs taken from heart tissue that are known for a lower recovery rate. Moreover the type of tissue biopsied may be crucial, with vegetations more likely than heart valves to yield a positive PCR product [36, 37]. Also differences in study design, method applied, and prolonged antibiotic treatment periods before surgery may explain our relatively lower broad-range PCR sensitivity compared to other studies. In fact, at least 93.8% of patients with negative broad-range PCR received antibiotics before surgery. Broad-range PCR test results can turn negative more quickly than histopathological or gross evidence of IE [38] which can lead to discrepancies between PCR and other diagnostic methods when applying nucleic amplification techniques. Nevertheless, the high positive predictive value of PCR in our study shows that broad-range PCR on resected heart tissue or swabs is a reliable method to diagnose IE in culture-negative patients as in 90.9% of all patients with positive test results it further confirmed the presence of IE, whereas the low negative predictive value of 14.3% indicates that a negative PCR result apparently cannot exclude IE.

For 4 patients in whom the diagnosis of IE could not be established by clinical evidence alone, broad-range PCR provided the etiologic diagnosis of IE, thereby confirming the suspicion of IE. If broad-range PCR had been primarily accepted as a specific diagnostic criterion, as proposed by some [19, 21, 24], these cases of “possible IE” would have been reclassified as “definite IE” which would have increased the sensitivity and positive predictive value of PCR in our investigation.

The majority of the patients with definite and possible IE of whom data of preBC were available (78.8%) had a positive blood culture (86.6%). In only 38.0% of these patients, broad-range PCR was positive. In one-third of these patients (*n* = 9) with a positive blood culture and a broad-range PCR, the microorganisms identified differed among both techniques in both the species and the genera. In two of these cases, blood culture revealed* S. alactolyticus* and* S. auricularis* which are not reported in literature to cause IE whereas broad-range PCR identified microorganisms (*S. gallolyticus* and* S. aureus*, resp.) consistent with microorganisms causing IE [39] pointing to possible misidentification or even contamination of blood cultures in these cases. In two other cases, broad-range PCR identified fastidious bacteria such as* Granulicatella adiacens* and the intracellular organism* Bartonella quintana* which can be easily missed by conventional culture-based methods [13, 36, 40]. In the latter case conventional culture revealed* S. epidermidis* which can be clearly assumed to be a contamination in the clinical context. For 2 cases in which blood culture revealed the genus only, broad-range PCR identified the species of the bacteria and thereby specified the etiology of the causative microorganisms. All these cases represent a possible diagnostic “add-on” of PCR diagnostics as the molecular identification of the causative pathogen by broad-range PCR is regarded more accurately than by blood culture and biochemical identification [41]. Furthermore, broad-range PCR from valve tissue is known to be more specific in the etiologic diagnosis of IE and, therefore, may also lead to better tailored treatment regimens after IE and cardiac surgery.

In the majority of blood culture-positive cases, broad-range PCR remained negative (62.0%) which can be ascribed either to prolonged preoperative antibiotic treatment as 97.7% of these patients received antibiotics before surgery or to possible inadequate intraoperative specimen collection. This is why several fractions of the valve tissue should best be collected for broad-range PCR [22, 36] and swabs should be avoided. In one case out of 11 blood culture-negative IE cases (9.1%), broad-range PCR provided the sole etiologic diagnosis of IE. Although in the routine clinical setting the sensitivity of PCR testing was somewhat lower than expected, our study confirms the usefulness of PCR amplification and sequencing of the bacterial 16S-rDNA as an important add-on technology in the analysis of resected heart valve tissue of swabs in cases with tissue or swab culture-negative IE as described previously [19, 21, 24, 28]. Our study identified a total of 20 patients (21.5%) who possibly profited from the application of broad-range PCR in case of heart tissue or swab culture-negative IE by the following: (i) reclassifying a “possible IE” to “definite IE” considering broad-range PCR as a criterion for IE (15.0%); (ii) providing the sole etiologic identification of the causative pathogen (25.0%); (iii) correction of genera and species of probably misidentified causative pathogens resulting from preBC diagnostics (35.0%); (iv) adding the correct species identification specification to the pathogen identified by preBC (20.0%); and (v) excluding possible contaminants resulting from preBC diagnostics (5.0%).

## 5. Conclusion

Novel perspectives on the management of IE are needed to decrease the rate of residual deaths by accelerating the processes of diagnosis, risk stratification, and facilitating the individualised adaption of antimicrobial therapy. In view of our results we, thus, consider broad-range PCR on resected heart valve tissue as a helpful supplementary tool in patients with culture-negative or questionable IE. Further research into the “added value” of broad-range PCR in the diagnosis of IE, compared to conventional diagnostics in routine clinical settings, as well as a better standardization and further improvements of PCR technology including increased sensitivity and specificity are necessary before this technique can be finally included in Duke's classification scheme. Nevertheless, culture-dependent methods will remain the mainstay in the diagnosis of IE as they offer the possibility of antimicrobial susceptibility testing of the causative pathogen.

## Figures and Tables

**Table 1:** Distribution of types of valves infected within the study group.

	Type of valve infected (*N* = 113^*∗*^)		
	Native	Prosthetic	Cardiac devices
Aortic	37	28	
Mitral	34	5	
Tricuspidal	2	1	
			6

*N* (%)	73 (64.6)	34 (30.1)	6 (5.3)

104 patients; F/M ratio 1 : 3; mean age 66.0 ± 12.6 years			

^*∗*^Heart valves of patients with two infected heart valves are counted separately.

**Table 2:** Overview of PCR results and preoperative antibiotic treatment. Crude numbers refer to number of patients; numbers in parentheses indicate the proportion of valve tissue samples versus swabs.

Tissue samples/swabs^1^				
		Number of patients	PCR	
			Positive	Negative
Total		**114**	**40 (28/12)**	**74 (42/32)**
Preoperative antibiotic treatment		101	33 ^*∗*^ND 7	68 ^*∗*^ND 4

IE	Definite	100	36 (25/11)	64 (35/29)
	Possible	4	4 (3/1)	0
	No evidence	10	0	10 (7/3)

^*∗*^ND: no data available; for 2 patients a preoperative antibiotic treatment could be excluded by medical chart.

^1^Positive tissue/swab cultures were available for 11 specimens that were exceptionally analyzed using broad-range PCR before a negative culture result was obtained. These data were described but not used for the computation of sensitivity, specificity, and positive and negative predictive values of broad-range PCR.

**Table 3:** Overview of species identification obtained with PCR and sequencing methods and comparison to the results of preoperative blood cultures (preBC).

PCR positive *n* = 40, species identification *n* = 33^*∗*^					
Concordance *n* = 9 (PCR/preBC^*∗∗*^)	*n*	Discordance *n* = 14 (on genus and/or species level)		Additional information *n* = 10 (no preBC data available)	*n*
		PCR	Result of preBC^*∗∗*^		
*Enterococcus faecalis*	5	*Enterococcus faecalis*	*Streptococcus gordonii*	*Enterococcus faecalis*	1
*Staphylococcus aureus*	1	*Enterococcus faecium*	*Enterococcus faecalis*	*Staphylococcus aureus*	2
*Streptococcus agalactiae*	1	*Staphylococcus aureus*	*Staphylococcus auricularis*	*Staphylococcus epidermidis*	1
*Aerococcus urinae*	1	*Staphylococcus epidermidis*	*Streptococcus oralis*	*Staphylococcus lugdunensis*	1
*Gemella haemolysans*	1	*Streptococcus gallolyticus *subsp. *pasteurianus*	*Streptococcus alactolyticus*	*Streptococcus mitis*	1
		*Streptococcus gordonii*	*Streptococcus pneumoniae*	*Streptococcus pneumoniae*	1
		*Streptococcus gordonii*	*Streptococcus viridans*	*Streptococcus sanguinis*	1
		*Streptococcus mitis*	*Streptococcus intermedius*	*Anoxybacillus *spp.	1
		*Bartonella quintana*	*Staphylococcus epidermidis*	*Propionibacterium acnes*	1
		*Granulicatella adiacens*	*Streptococcus viridans*		
		*Streptococcus oralis*	*Streptococcus viridans*		
		*Enterococcus faecium*	Gram-positive cocci		
		*Staphylococcus epidermidis*	Gram-positive cocci		
		*Propionibacterium acnes*	Negative preBC		

^*∗*^In 7 patients with positive PCR amplicon, direct sequencing failed due to a bacterial mixture *n* = 4 or low DNA concentrations *n* = 3.

^*∗∗*^Results of preBC were taken and analyzed in hospitals of primary care according to local standard microbiological laboratory procedures.

**Table 4:** Comparison of PCR with results of tissue or swab culture. For a small patient group (*n* = 11) results of both methods (PCR amplification and culture of swab or tissue) were exceptionally gained upon special clinical request. The direct comparison is provided in this table; these data were not used for further computation of sensitivity, specificity, and positive and negative predictive values of broad-range PCR.

Result	Culture	PCR
Positive in both methods, concordance on species level *n* = 5	3 ×* Enterococcus faecalis*	
	*Staphylococcus epidermidis*	
	*Staphylococcus lugdunensis*	

Positive but discordant *n* = 2	*Staphylococcus epidermidis*	*Streptococcus gordonii*
	*Staphylococcus pasteuri*	*Streptococcus gallolyticus*

Culture positive but PCR amplification negative *n* = 4	2 ×* Enterococcus faecium*	neg
	*Staphylococcus aureus*	neg
	Coagulase-negative staphylococci	neg

**Table 5:** Frequencies and etiology of causative pathogens identified by broad-range PCR in tissue or swab culture-negative IE patients (*n* = 33).

Causative pathogens identified by PCR in culture-negative IE	*n*	%
*Aerococcus urinae*	1	3.0
*Anoxybacillus *spp.	1	3.0
*Bartonella quintana*	1	3.0
Enterococci	6	18.2
*Gemella haemolysans*	1	3.0
*Granulicatella adiacens*	1	3.0
*Propionibacterium acnes*	2	6.1
*Staphylococcus aureus*	4	12.1
*Staphylococcus epidermidis*	2	6.1
Streptococci	7	21.2
No sequencing possible	7	21.2

Total	33	100

**Table 6:** Calculation of sensitivity, specificity, and positive and negative predictive values. Calculation of sensitivity, specificity, and positive and negative predictive values is based on a dataset of *n* = 103. The gold standard is defined according to the criteria outlined in Definition of Diagnosed IE (Section 2).

		Diagnosis			
		+	−		
**PCR**	+	*30*	*3*	**90.9%**	Positive predictive value
	−	*60*	*10*	**14.3%**	Negative predictive value
		**33.3%**	**76.9%**		
		Sensitivity	Specificity		
